# Range expansion of Bombus (Pyrobombus) bimaculatus Cresson in Canada (Hymenoptera, Apidae)

**DOI:** 10.3897/BDJ.11.e104657

**Published:** 2023-05-31

**Authors:** Cory Sheffield, Kirsten M. Palmier

**Affiliations:** 1 Royal Saskatchewan Museum, Regina, Canada Royal Saskatchewan Museum Regina Canada; 2 Department of Biology, University of Regina, Regina, Canada Department of Biology, University of Regina Regina Canada

**Keywords:** two-spotted Bumble Bee, iNaturalist, Prairies Ecozone, Atlantic Maritime Ecozone

## Abstract

**Background:**

The two-spotted bumble bee, *Bombusbimaculatus* Cresson, 1863 (Hymenoptera, Apidae), is a common species in central North America, with few published records of this species in Canada west of Ontario or east of Quebec.

**New information:**

Based on recently collected specimens from Saskatchewan and confirmed records posted to iNaturalist (https://www.inaturalist.org/) in the past 10 years (i.e. since 2013), we provide evidence that this species has only recently expanded its range in Canada, westwards into the Prairies Ecozone (Manitoba, Saskatchewan) and east into the Maritime Provinces (New Brunswick, Nova Scotia, Prince Edward Island).

## Introduction

Bumble bees are amongst the most familiar of insects and have been the subject of natural history investigation for centuries. There have been several historic treatments of bumble bee taxonomy and distribution in North America, but Williams et al. (2014) provide the most detailed and recent information on species distribution in North America. Since then, recent additions to the North American fauna include *Bombuskluanensis* Williams and Cannings, 2016 (Williams et al. 2016), a taxonomic treatment of the *B.bifarius* Cresson, 1878 complex resulting in the recognition of two species, *B.bifarius* and *B.vancouverensis* Cresson, 1878 (Ghisbain et al. 2020) and the recognition of *B.johanseni* Sladen, 1919 as a valid species (Sheffield et al. 2020), first shown by Martinet et al. (2019) who described it as a new taxon, *B.interacti* Martinet, Brasero and Rasmont, 2019. With respect to the bumble bees of Canada, Curry (1984), Neave (1933) and Laverty and Harder (1988) provided keys to the species of Saskatchewan, Manitoba and eastern Canada, respectively; Buckell (1951), Cannings (2011) and Sheffield and Heron (2019) summarised species in British Columbia; Sheffield et al. (2014) and Gibbs et al. (2023) reviewed the species of the Canadian Prairie Provinces and Manitoba, respectively. Other important works with coverage of species occurring in Canada include Stephen (1957) for the west, Plath (1934) and Mitchell (1962) for the east and the more general works of Cresson (1863), Franklin (1913), Frison (1923), Frison (1926) and Milliron (Milliron 1971, Milliron 1973a, Milliron 1973b).

*Bombusbimaculatus* Cresson was described in 1863 from material from Connecticut (Cresson 1863), with synonymous species described from West Virginia (Cresson 1878) and Massachussettes (Bequaert and Plath 1925). In an early comprehensive treatment of New World bumble bees, Franklin (1913) commented that *B.bimaculatus* was very rare in south-eastern Canada, occurring mostly in the United States from New England as far west as eastern Nebraska. Plath (1934) reviewed the species of eastern North America and depicted *B.bimaculatus* more extensively distributed in Canada, ranging from eastern Ontario (including just north of Lake Superior), southern Quebec and perhaps New Brunswick and throughout all of the eastern United States as far west as North Dakota, South Dakota, Nebraska and Kansas. Laverty and Harder (1988) also recorded *B.bimaculatus* Cresson from southern Ontario and Quebec though not New Brunswick and Nova Scotia despite the earlier reports of Boulanger et al. (1967) and Vander Kloet (1977), respectively, suggesting that this species was mostly known from the Mixedwood Plains Ecozone in Canada and, perhaps, into bordering locations in the Boreal Ecozone. It was not known from Manitoba (Neave 1933, Turnock et al. 2006) until first reported by Sheffield et al. (2014) from specimens identifed by CSS that were collected in 2009; these records were subsequently included in Williams et al. (2014). Curry (1984) did not report this species from Saskatchewan and, more recently, this species was not recorded from Alberta (Prescott et al. 2019). Subsequent works in Nova Scotia (Sheffield et al. 2003, Sheffield et al. 2009, Sheffield et al. 2013) also did not include *B.bimaculatus*, though it was recently re-confirmed in New Brunswick (Brooks and Nocera 2020), based on iNaturalist observations and Cape Breton Island, Nova Scotia (Dominey 2021, Kosick 2021, Kosick 2021).

In the United States, Franklin (1913) and LaBerge and Webb (1962) also reported this species from Nebraska, but the latter indicated that it was primarily an eastern species with relatively few numbers from the eastern part of the State. Other records from the western part of its range in the United States include Franklin (1913) and Frison (1923) from Kansas, Franklin (1913), Frison (1926), Medler and Carney (1963), Colla et al. (2011) from Minnesota and Frison (1926) from South Dakota; Koch et al. (2015) included only eight records from North Dakota, South Dakota and Nebraska. This species was not included in any of the treatments for the western North America (Stephen 1957, Koch et al. 2012), but was recorded for the first time from eastern Montana in 2017 (Dolan et al. 2017). Lozier et al. (2011), Colla et al. (2012) and Jacobson et al. (2018) all indicated that its populations were stable to increasing in North America.

In the last decade, data from both active sampling and online databases have accumulated, suggesting that this species' range has been spreading in Canada and also westwards into the United States. Our purpose is to provide documentation that support that this species is now much more widespread in North America than recent treatments (i.e. Williams et al. (2014)) account for.

## Materials and methods

A database containing 34,802 [modified to remove observations without latitude and longitude coordinates and dates] North American occurrence records determined as *Bombusbimaculatus* was downloaded from the Global Biodiversity Information Facility (GBIF); additional specimens from the dataset of Koch et al. (2015). The GBIF data also contained "Research Grade" observations from iNaturalist (https://www.inaturalist.org/). The specimen determinations were accepted as is, though some specimens from the western United States pre-2000 (i.e. Arizona, Colorado, Montana and Oregon) are likely questionable, based on past (i.e. Titus (1902), Franklin (1913), Frison (1923), Frison (1926), Stephen (1957), Medler and Carney (1963), Thorp et al. (1983)) and more recent taxonomic treatments (Koch et al. 2012, Williams et al. 2014). No records from these States were included in the recent database of Koch et al. (2015). We acknowledge that there are likely misidentifications in these datasets, though we feel that these do not impact the over patterns presented in this paper for documenting the range and spread of *B.bimaculatus*.

Additional records from Saskatchewan (see Materials below) from iNaturalist and the Royal Saskatchewan Museum (RSKM), Regina, Saskatchewan were also included. The datasets were cleaned and harmonised using R (v.4.0.3, R Core Team (2022)) and the tidyverse package (v.1.3.0, Wickham (2019)). Visualisation and mapping were done using the ggplot2 package (v.3.3.3, Wickham (2016)) and raster package (v.3.6-20, Hijmans (2023)).

## Data resources

The North American dataset used here for mapping was downloaded from GBIF.org (2023). The dataset contained records dating back to 1885.

## Taxon treatments

### Bombus (Pyrobombus) bimaculatus

Cresson, 1863

EADFFAB5-78F6-5101-A69D-5103B0DCA1AA

*Bombusbimaculatus* Cresson, 1863 - Cresson (1863): 92 [♂]. **Holotype** ♂. USA, Connecticut, by E. Norton [ANSP no. 2715].
*Bombusridingsii* Cresson, 1878 - Cresson (1878): 182 [♀]. Synonymy by Burks (1951): 1251, as a variety; synonymy by Mitchell (1962): 531. **Syntypes** ♀♀. USA, West Virginia, by J. Ridings [ANSP no. 2635].
Bremusbimaculatusvar.ahenus Bequaert and Plath, 1925 - Bequaert and Plath (1925): 275 [♀,♂]. Synonymy by Hurd (1979): 2198. **Holotype** ♀. USA, Massachusetts, Forest Hills, by O.E. Plath [MCZ no. 15,281].
Bremusbimaculatusvar.arboreti Bequaert and Plath, 1925 - Bequaert and Plath (1925): 276 [♀]. Synonymy by Hurd (1979): 2198. **Holotype** ♀. USA, Massachusetts, Forest Hills, on 2 June 1924, by O.E. Plath [MCZ no. 15,282].

#### Materials

- **Type status:** Other material. **Occurrence:** occurrenceDetails: iNaturalist observation; catalogNumber: 13835405; occurrenceRemarks: Research Grade; recordedBy: djsheffield; individualCount: 1; lifeStage: adult; associatedMedia: https://static.inaturalist.org/photos/20417139/medium.jpg?1530122554; occurrenceID: 3AD960DF-5B0E-50D8-A677-9599386519DD; **Taxon:** scientificName: Bombusbimaculatus; kingdom: Animalia; phylum: Arthropoda; class: Insecta; order: Hymenoptera; family: Apidae; genus: Bombus; subgenus: Pyrobombus; **Location:** country: Canada; stateProvince: Saskatchewan; locality: Wascana Centre, Regina, SK, CA; decimalLatitude: 50.4395783; decimalLongitude: -104.6165362; coordinateUncertaintyInMeters: 5; **Event:** samplingProtocol: none specified; eventDate: 06/27/2018; **Record Level:** source: https://www.inaturalist.org/observations/13835405
- **Type status:** Other material. **Occurrence:** occurrenceDetails: iNaturalist observation; catalogNumber: 14775836; occurrenceRemarks: Research Grade; recordedBy: cory_silas_sheffield; individualCount: 1; lifeStage: adult; associatedMedia: https://static.inaturalist.org/photos/22048949/medium.jpg?1532664693; occurrenceID: 9E2AAA8C-90A5-585E-A31A-43B6BDE39E7A; **Taxon:** scientificName: Bombusbimaculatus; kingdom: Animalia; phylum: Arthropoda; class: Insecta; order: Hymenoptera; family: Apidae; genus: Bombus; subgenus: Pyrobombus; **Location:** country: Canada; stateProvince: Saskatchewan; locality: Victoria Ave, Regina, SK, CA; decimalLatitude: 50.44732833; decimalLongitude: -104.6147217; coordinateUncertaintyInMeters: 10; **Event:** samplingProtocol: none specified; eventDate: 07/26/2018; **Record Level:** source: https://www.inaturalist.org/observations/14775836
- **Type status:** Other material. **Occurrence:** occurrenceDetails: iNaturalist observation; catalogNumber: 14984878; occurrenceRemarks: Research Grade; recordedBy: cory_silas_sheffield; individualCount: 1; lifeStage: adult; associatedMedia: https://static.inaturalist.org/photos/22352937/medium.jpg?1533046158; occurrenceID: 8961341F-9246-536D-B163-7EB7CE1CC7A0; **Taxon:** scientificName: Bombusbimaculatus; kingdom: Animalia; phylum: Arthropoda; class: Insecta; order: Hymenoptera; family: Apidae; genus: Bombus; subgenus: Pyrobombus; **Location:** country: Canada; stateProvince: Saskatchewan; locality: Wascana Centre, Regina, SK, CA; decimalLatitude: 50.43961333; decimalLongitude: -104.6165383; coordinateUncertaintyInMeters: 5; **Event:** samplingProtocol: none specified; eventDate: 07/29/2018; **Record Level:** source: https://www.inaturalist.org/observations/14984878
- **Type status:** Other material. **Occurrence:** occurrenceDetails: iNaturalist observation; catalogNumber: 17006931; occurrenceRemarks: Research Grade; recordedBy: cory_silas_sheffield; individualCount: 1; lifeStage: adult; associatedMedia: https://static.inaturalist.org/photos/25717415/medium.jpg?1538165406; occurrenceID: C033564E-4784-5FFC-8601-D9D4C3C5C3C3; **Taxon:** scientificName: Bombusbimaculatus; kingdom: Animalia; phylum: Arthropoda; class: Insecta; order: Hymenoptera; family: Apidae; genus: Bombus; subgenus: Pyrobombus; **Location:** country: Canada; stateProvince: Saskatchewan; locality: Victoria Ave, Regina, SK, CA; decimalLatitude: 50.44732833; decimalLongitude: -104.6147217; coordinateUncertaintyInMeters: 10; **Event:** samplingProtocol: none specified; eventDate: 07/26/2018; **Record Level:** source: https://www.inaturalist.org/observations/17006931
- **Type status:** Other material. **Occurrence:** occurrenceDetails: iNaturalist observation; catalogNumber: 17006945; occurrenceRemarks: Research Grade; recordedBy: cory_silas_sheffield; individualCount: 1; lifeStage: adult; associatedMedia: https://static.inaturalist.org/photos/25717437/medium.jpg?1538165440; occurrenceID: 7ACD218F-B713-5CF4-968E-0AF2F4C76847; **Taxon:** scientificName: Bombusbimaculatus; kingdom: Animalia; phylum: Arthropoda; class: Insecta; order: Hymenoptera; family: Apidae; genus: Bombus; subgenus: Pyrobombus; **Location:** country: Canada; stateProvince: Saskatchewan; locality: Retallack St, Regina, SK, CA; decimalLatitude: 50.44202833; decimalLongitude: -104.6226967; coordinateUncertaintyInMeters: 5; **Event:** samplingProtocol: none specified; eventDate: 07/28/2018; **Record Level:** source: https://www.inaturalist.org/observations/17006945
- **Type status:** Other material. **Occurrence:** occurrenceDetails: iNaturalist observation; catalogNumber: 17006989; occurrenceRemarks: Research Grade; recordedBy: cory_silas_sheffield; individualCount: 1; lifeStage: adult; associatedMedia: https://static.inaturalist.org/photos/25717504/medium.jpg?1538165501; occurrenceID: 4467D420-E26D-5199-9811-48E2AD75E598; **Taxon:** scientificName: Bombusbimaculatus; kingdom: Animalia; phylum: Arthropoda; class: Insecta; order: Hymenoptera; family: Apidae; genus: Bombus; subgenus: Pyrobombus; **Location:** country: Canada; stateProvince: Saskatchewan; locality: Victoria Ave, Regina, SK, CA; decimalLatitude: 50.44744167; decimalLongitude: -104.6139667; coordinateUncertaintyInMeters: 10; **Event:** samplingProtocol: none specified; eventDate: 07/26/2018; **Record Level:** source: https://www.inaturalist.org/observations/17006989
- **Type status:** Other material. **Occurrence:** occurrenceDetails: iNaturalist observation; catalogNumber: 25003160; occurrenceRemarks: Research Grade; recordedBy: cory_silas_sheffield; individualCount: 1; lifeStage: adult; associatedMedia: https://static.inaturalist.org/photos/38686785/medium.jpg?1557678418; occurrenceID: 29EEC42D-273F-5E08-950C-9AA43F328DDE; **Taxon:** scientificName: Bombusbimaculatus; kingdom: Animalia; phylum: Arthropoda; class: Insecta; order: Hymenoptera; family: Apidae; genus: Bombus; subgenus: Pyrobombus; **Location:** country: Canada; stateProvince: Saskatchewan; locality: Retallack St, Regina, SK, CA; decimalLatitude: 50.4419844; decimalLongitude: -104.6227672; coordinateUncertaintyInMeters: 22; **Event:** samplingProtocol: none specified; eventDate: 05/12/2019; **Record Level:** source: https://www.inaturalist.org/observations/25003160
- **Type status:** Other material. **Occurrence:** occurrenceDetails: iNaturalist observation; catalogNumber: 25679542; occurrenceRemarks: Research Grade; recordedBy: sarah_306; individualCount: 1; lifeStage: adult; associatedMedia: https://static.inaturalist.org/photos/39801310/medium.jpeg?1558714628; occurrenceID: 67452284-EDBD-5AA0-B315-F0F8E76C3A66; **Taxon:** scientificName: Bombusbimaculatus; kingdom: Animalia; phylum: Arthropoda; class: Insecta; order: Hymenoptera; family: Apidae; genus: Bombus; subgenus: Pyrobombus; **Location:** country: Canada; stateProvince: Saskatchewan; locality: Sherwood Dr @ Edward St (EB), Regina, CA; decimalLatitude: 50.47503129; decimalLongitude: -104.6429203; coordinateUncertaintyInMeters: 9; georeferenceSources: gps; **Event:** samplingProtocol: none specified; eventDate: 05/23/2019; **Record Level:** source: https://www.inaturalist.org/observations/25679542
- **Type status:** Other material. **Occurrence:** occurrenceDetails: iNaturalist observation; catalogNumber: 27533496; occurrenceRemarks: Research Grade; recordedBy: cory_silas_sheffield; individualCount: 1; lifeStage: adult; associatedMedia: https://static.inaturalist.org/photos/42848942/medium.jpg?1561328866; occurrenceID: F5773232-308B-5150-950B-17BF040C12EB; **Taxon:** scientificName: Bombusbimaculatus; kingdom: Animalia; phylum: Arthropoda; class: Insecta; order: Hymenoptera; family: Apidae; genus: Bombus; subgenus: Pyrobombus; **Location:** country: Canada; stateProvince: Saskatchewan; locality: Cameron St, Regina, SK, CA; decimalLatitude: 50.43555833; decimalLongitude: -104.6255633; coordinateUncertaintyInMeters: 5; **Event:** samplingProtocol: none specified; eventDate: 06/23/2019; **Record Level:** source: https://www.inaturalist.org/observations/27533496
- **Type status:** Other material. **Occurrence:** occurrenceDetails: iNaturalist observation; catalogNumber: 28411439; occurrenceRemarks: Research Grade; recordedBy: meghanmickelson; individualCount: 1; lifeStage: adult; associatedMedia: https://static.inaturalist.org/photos/44288668/medium.jpeg?1562554211; occurrenceID: C2BFC522-0A1B-5C03-AF39-B27B612813D4; **Taxon:** scientificName: Bombusbimaculatus; kingdom: Animalia; phylum: Arthropoda; class: Insecta; order: Hymenoptera; family: Apidae; genus: Bombus; subgenus: Pyrobombus; **Location:** country: Canada; stateProvince: Saskatchewan; locality: Buena Vista, SK, CA; decimalLatitude: 52.10832152; decimalLongitude: -106.6688233; coordinateUncertaintyInMeters: 55; **Event:** samplingProtocol: none specified; eventDate: 07/07/2019; **Record Level:** source: https://www.inaturalist.org/observations/28411439
- **Type status:** Other material. **Occurrence:** occurrenceDetails: iNaturalist observation; catalogNumber: 29798922; occurrenceRemarks: Research Grade; recordedBy: cory_silas_sheffield; individualCount: 1; lifeStage: adult; associatedMedia: https://static.inaturalist.org/photos/46524789/medium.jpg?1564423555; occurrenceID: 1943D767-8FA1-5132-BAFC-B21E50038135; **Taxon:** scientificName: Bombusbimaculatus; kingdom: Animalia; phylum: Arthropoda; class: Insecta; order: Hymenoptera; family: Apidae; genus: Bombus; subgenus: Pyrobombus; **Location:** country: Canada; stateProvince: Saskatchewan; locality: 15th Ave, Regina, SK, CA; decimalLatitude: 50.44233; decimalLongitude: -104.6205833; coordinateUncertaintyInMeters: 10; **Event:** samplingProtocol: none specified; eventDate: 07/29/2019; **Record Level:** source: https://www.inaturalist.org/observations/29798922
- **Type status:** Other material. **Occurrence:** occurrenceDetails: iNaturalist observation; catalogNumber: 29802698; occurrenceRemarks: Research Grade; recordedBy: djsheffield; individualCount: 1; lifeStage: adult; associatedMedia: https://static.inaturalist.org/photos/46530766/medium.jpg?1564426665; occurrenceID: 46EEAE46-E777-5CA1-A497-8B91D4CD2451; **Taxon:** scientificName: Bombusbimaculatus; kingdom: Animalia; phylum: Arthropoda; class: Insecta; order: Hymenoptera; family: Apidae; genus: Bombus; subgenus: Pyrobombus; **Location:** country: Canada; stateProvince: Saskatchewan; locality: 15th Ave, Regina, SK, CA; decimalLatitude: 50.44228183; decimalLongitude: -104.6206921; coordinateUncertaintyInMeters: 12; **Event:** samplingProtocol: none specified; eventDate: 07/29/2019; **Record Level:** source: https://www.inaturalist.org/observations/29802698
- **Type status:** Other material. **Occurrence:** occurrenceDetails: iNaturalist observation; catalogNumber: 29806723; occurrenceRemarks: Research Grade; recordedBy: cory_silas_sheffield; individualCount: 1; lifeStage: adult; associatedMedia: https://static.inaturalist.org/photos/46537382/medium.jpeg?1564429997; occurrenceID: 23AB240B-EFC4-5ED7-86DF-FB4059F57D0C; **Taxon:** scientificName: Bombusbimaculatus; kingdom: Animalia; phylum: Arthropoda; class: Insecta; order: Hymenoptera; family: Apidae; genus: Bombus; subgenus: Pyrobombus; **Location:** country: Canada; stateProvince: Saskatchewan; locality: Cathedral, Regina, SK, Canada; decimalLatitude: 50.44418732; decimalLongitude: -104.6196801; coordinateUncertaintyInMeters: 122; **Event:** samplingProtocol: none specified; eventDate: 07/29/2019; **Record Level:** source: https://www.inaturalist.org/observations/29806723
- **Type status:** Other material. **Occurrence:** occurrenceDetails: iNaturalist observation; catalogNumber: 30221212; occurrenceRemarks: Research Grade; recordedBy: sarah_306; individualCount: 1; lifeStage: adult; associatedMedia: https://static.inaturalist.org/photos/47204264/medium.jpeg?1564974488; occurrenceID: 375DD05A-DDC2-5B50-82C4-BF9A1268E32A; **Taxon:** scientificName: Bombusbimaculatus; kingdom: Animalia; phylum: Arthropoda; class: Insecta; order: Hymenoptera; family: Apidae; genus: Bombus; subgenus: Pyrobombus; **Location:** country: Canada; stateProvince: Saskatchewan; locality: Regina, SK, Canada; decimalLatitude: 50.47494507; decimalLongitude: -104.6425247; georeferenceSources: gps; **Event:** samplingProtocol: none specified; eventDate: 08/04/2019; **Record Level:** source: https://www.inaturalist.org/observations/30221212
- **Type status:** Other material. **Occurrence:** occurrenceDetails: iNaturalist observation; catalogNumber: 30221964; occurrenceRemarks: Research Grade; recordedBy: latebloomer2; individualCount: 1; lifeStage: adult; associatedMedia: https://static.inaturalist.org/photos/47205538/medium.jpg?1564975375; occurrenceID: 40D0A612-FB4A-5BDC-8F7C-7951CF04C3D5; **Taxon:** scientificName: Bombusbimaculatus; kingdom: Animalia; phylum: Arthropoda; class: Insecta; order: Hymenoptera; family: Apidae; genus: Bombus; subgenus: Pyrobombus; **Location:** country: Canada; stateProvince: Saskatchewan; locality: Kiwanis Waterfall Park, Regina, SK, CA; decimalLatitude: 50.43558528; decimalLongitude: -104.6219462; coordinateUncertaintyInMeters: 1273; **Event:** samplingProtocol: none specified; eventDate: 08/04/2019; **Record Level:** source: https://www.inaturalist.org/observations/30221964
- **Type status:** Other material. **Occurrence:** occurrenceDetails: iNaturalist observation; catalogNumber: 30584482; occurrenceRemarks: Research Grade; recordedBy: cory_silas_sheffield; individualCount: 1; lifeStage: adult; associatedMedia: https://static.inaturalist.org/photos/47789579/medium.jpg?1565488686; occurrenceID: BA9FAE23-C0F8-5D00-9472-1ED212505ADB; **Taxon:** scientificName: Bombusbimaculatus; kingdom: Animalia; phylum: Arthropoda; class: Insecta; order: Hymenoptera; family: Apidae; genus: Bombus; subgenus: Pyrobombus; **Location:** country: Canada; stateProvince: Saskatchewan; locality: Retallack St, Regina, SK, CA; decimalLatitude: 50.44203; decimalLongitude: -104.623; coordinateUncertaintyInMeters: 5; **Event:** samplingProtocol: none specified; eventDate: 08/10/2019; **Record Level:** source: https://www.inaturalist.org/observations/30584482
- **Type status:** Other material. **Occurrence:** occurrenceDetails: iNaturalist observation; catalogNumber: 30880573; occurrenceRemarks: Research Grade; recordedBy: djsheffield; individualCount: 1; lifeStage: adult; associatedMedia: https://static.inaturalist.org/photos/48274049/medium.jpg?1565887254; occurrenceID: E47B0996-A460-5715-AD8A-08CC737FFB93; **Taxon:** scientificName: Bombusbimaculatus; kingdom: Animalia; phylum: Arthropoda; class: Insecta; order: Hymenoptera; family: Apidae; genus: Bombus; subgenus: Pyrobombus; **Location:** country: Canada; stateProvince: Saskatchewan; locality: Retallack St, Regina, SK, CA; decimalLatitude: 50.44199455; decimalLongitude: -104.6229456; coordinateUncertaintyInMeters: 22; **Event:** samplingProtocol: none specified; eventDate: 08/15/2019; **Record Level:** source: https://www.inaturalist.org/observations/30880573
- **Type status:** Other material. **Occurrence:** occurrenceDetails: iNaturalist observation; catalogNumber: 30940499; occurrenceRemarks: Research Grade; recordedBy: sarah_306; individualCount: 1; lifeStage: adult; associatedMedia: https://static.inaturalist.org/photos/48373636/medium.jpeg?1565974844; occurrenceID: A85B689F-55BB-562B-9DC3-AAA115907FEA; **Taxon:** scientificName: Bombusbimaculatus; kingdom: Animalia; phylum: Arthropoda; class: Insecta; order: Hymenoptera; family: Apidae; genus: Bombus; subgenus: Pyrobombus; **Location:** country: Canada; stateProvince: Saskatchewan; locality: Sherwood Dr @ Edward St (EB), Regina, SK, CA; decimalLatitude: 50.4749527; decimalLongitude: -104.6425705; georeferenceSources: gps; **Event:** samplingProtocol: none specified; eventDate: 08/16/2019; **Record Level:** source: https://www.inaturalist.org/observations/30940499
- **Type status:** Other material. **Occurrence:** occurrenceDetails: iNaturalist observation; catalogNumber: 46539545; occurrenceRemarks: Research Grade; recordedBy: cwf_erin; individualCount: 1; lifeStage: adult; associatedMedia: https://static.inaturalist.org/photos/73813750/medium.jpeg?1589928189; occurrenceID: 76853144-ED68-5D71-A769-2F6CF2895C32; **Taxon:** scientificName: Bombusbimaculatus; kingdom: Animalia; phylum: Arthropoda; class: Insecta; order: Hymenoptera; family: Apidae; genus: Bombus; subgenus: Pyrobombus; **Location:** country: Canada; stateProvince: Saskatchewan; locality: Argyle St N, Regina, SK, CA; decimalLatitude: 50.4806438; decimalLongitude: -104.6316069; coordinateUncertaintyInMeters: 161; **Event:** samplingProtocol: none specified; eventDate: 05/14/2020; **Record Level:** source: https://www.inaturalist.org/observations/46539545
- **Type status:** Other material. **Occurrence:** occurrenceDetails: iNaturalist observation; catalogNumber: 46786006; occurrenceRemarks: Research Grade; recordedBy: kpalmier; individualCount: 1; lifeStage: adult; associatedMedia: https://static.inaturalist.org/photos/74198671/medium.jpg?1590104851; occurrenceID: 8216655F-ABC7-5433-B536-D6F0FC882093; **Taxon:** scientificName: Bombusbimaculatus; kingdom: Animalia; phylum: Arthropoda; class: Insecta; order: Hymenoptera; family: Apidae; genus: Bombus; subgenus: Pyrobombus; **Location:** country: Canada; stateProvince: Saskatchewan; locality: Montague St, Regina, SK, CA; decimalLatitude: 50.44485398; decimalLongitude: -104.6302722; coordinateUncertaintyInMeters: 4; **Event:** samplingProtocol: none specified; eventDate: 05/21/2020; **Record Level:** source: https://www.inaturalist.org/observations/46786006
- **Type status:** Other material. **Occurrence:** occurrenceDetails: iNaturalist observation; catalogNumber: 51695891; occurrenceRemarks: Research Grade; recordedBy: cory_silas_sheffield; individualCount: 1; lifeStage: adult; associatedMedia: https://static.inaturalist.org/photos/82150777/medium.jpg?1593701998; occurrenceID: 1A6DBB21-AD3A-50CC-B0A3-DB0EB0A2BF15; **Taxon:** scientificName: Bombusbimaculatus; kingdom: Animalia; phylum: Arthropoda; class: Insecta; order: Hymenoptera; family: Apidae; genus: Bombus; subgenus: Pyrobombus; **Location:** country: Canada; stateProvince: Saskatchewan; locality: Wascana Creek, Regina, SK, CA; decimalLatitude: 50.43746667; decimalLongitude: -104.6281283; coordinateUncertaintyInMeters: 6; **Event:** samplingProtocol: none specified; eventDate: 07/02/2020; **Record Level:** source: https://www.inaturalist.org/observations/51695891
- **Type status:** Other material. **Occurrence:** catalogNumber: RSKM_ENT_E-226173; recordedBy: Ryan Oram; individualCount: 1; lifeStage: adult; occurrenceID: 87A39022-0660-5129-A358-DCF22F96445F; **Taxon:** scientificName: Bombusbimaculatus; kingdom: Animalia; phylum: Arthropoda; class: Insecta; order: Hymenoptera; family: Apidae; genus: Bombus; subgenus: Pyrobombus; **Location:** country: Canada; stateProvince: Saskatchewan; locality: Avonlea, SK, CA; decimalLatitude: 50.0246; decimalLongitude: -104.9831; **Event:** samplingProtocol: netting; eventDate: 06/18/2019
- **Type status:** Other material. **Occurrence:** catalogNumber: RSKM_ENT_E-189556; recordedBy: Kirsten Palmier; individualCount: 1; lifeStage: adult; occurrenceID: 88EB1216-984F-5531-A06B-2A5D4BE3D3EA; **Taxon:** scientificName: Bombusbimaculatus; kingdom: Animalia; phylum: Arthropoda; class: Insecta; order: Hymenoptera; family: Apidae; genus: Bombus; subgenus: Pyrobombus; **Location:** country: Canada; stateProvince: Saskatchewan; locality: Estevan, SK, CA; decimalLatitude: 49.1475; decimalLongitude: -102.9673; **Event:** samplingProtocol: Blue vane trap; startDayOfYear: 07/05/2018; endDayOfYear: 08/03/2018
- **Type status:** Other material. **Occurrence:** catalogNumber: RSKM_ENT_E-217788; recordedBy: Kirsten Palmier; individualCount: 1; lifeStage: adult; occurrenceID: 780F0200-5D87-5A96-B048-5D06D7466B98; **Taxon:** scientificName: Bombusbimaculatus; kingdom: Animalia; phylum: Arthropoda; class: Insecta; order: Hymenoptera; family: Apidae; genus: Bombus; subgenus: Pyrobombus; **Location:** country: Canada; stateProvince: Saskatchewan; locality: Assiniboia, SK, CA; decimalLatitude: 49.6343; decimalLongitude: -105.9931; **Event:** samplingProtocol: Blue vane trap; startDayOfYear: 07/27/2018; endDayOfYear: 08/14/2018

#### Distribution

*Bombusbimaculatus* is primarily a species of the eastern and central United States and Ontario and Quebec in Canada (Colla et al. 2011, Williams et al. 2014, Koch et al. 2015) (Fig. 1a, bFig. 1cFig. 1c). Since the publication of the most recent taxonomic treatment for North America (Williams et al. 2014), its range has spread into the Maritime Provinces (New Brunswick, Nova Scotia, Prince Edward Island) of Canada and northwestwards into southern Saskatchewan (Fig. 1c) and the adjacent United States.

## Discussion

**Figure 2.**
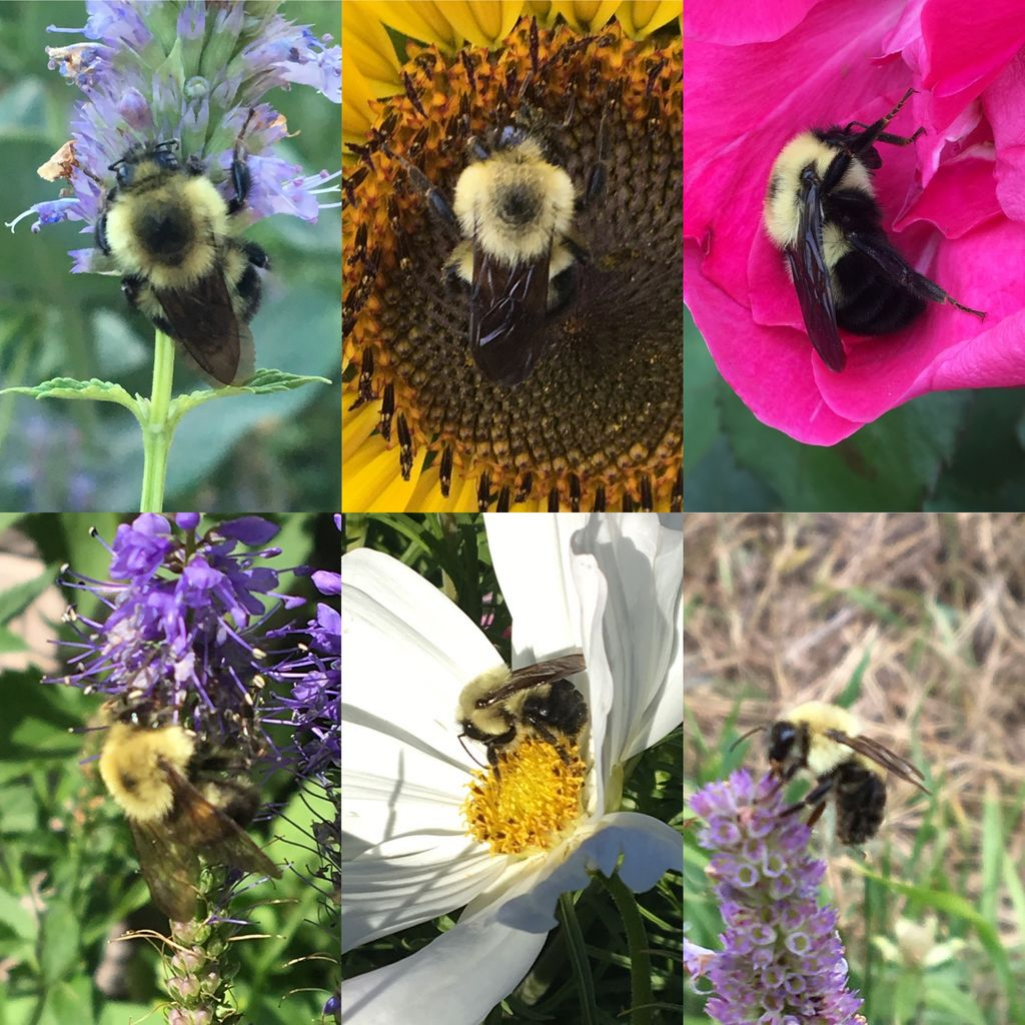
Bombus (Pyrobombus) bimaculatus Cresson specimens from Regina, Saskatchewan, Canada. Photos by Cory S. Sheffield.

**Figure 3.**
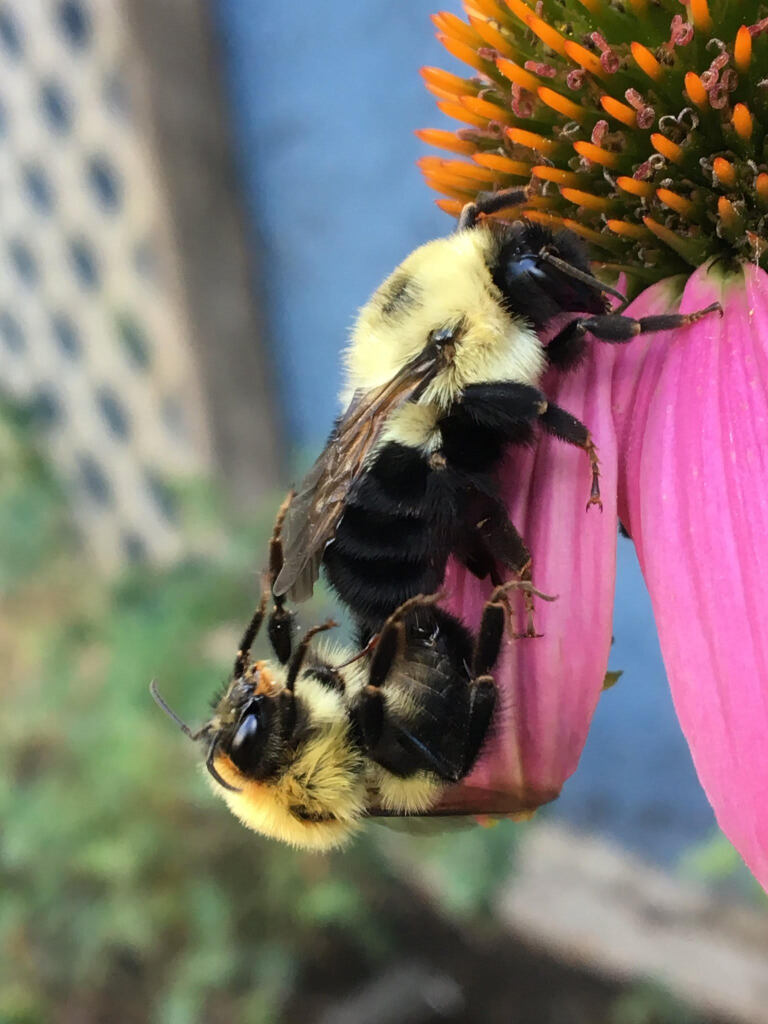
Bombus (Pyrobombus) bimaculatus Cresson mating; Regina, Saskatchewan, Canada. Photos by Cory S. Sheffield.

The recent spread of *B.bimaculatus* into western Canada, including Manitoba (Sheffield et al. 2014, Williams et al. 2014, Gibbs et al. 2023), Saskatchewan (Figs 2, 3) and the Maritime Provinces (Brooks and Nocera 2020, Dominey 2021, Kosick 2021, Kosick 2021, see Fig. 1) is seemingly by natural means as there are no confirmed records of this species being managed in any way that would promote its range expansion artificially. The oldest record from the Maritime Provinces on iNaturalist is from 2013 (i.e. just pre-Williams et al. (2014), though that work was likely in press at the time) from New Brunswick (Fig. 1b) and, at present, about 300 observations exist supporting that the species now widespread in that Province. The first record from Nova Scotia came three years later (i.e. 2016) and within two years, it was also widespread, now totalling ca. 400 observations. Establishment on Prince Edward Island has been a little slower, with only 39 observations since it was first detected on iNaturalist in 2019.

Kerr et al. (2015), Soroye et al. (2020) and others have indicated that several species of North American bumble bees are likely undergoing range declines in response to climate change, as has happened for some European species (see summary in Rasmont et al. (2021)), though *B.bimaculatus* may be demonstrating the opposite trend (see Jacobson et al. (2018)) as it has established in the Canadian Prairies and Maritime Provinces since 2009 (Manitoba) and post 2013 (i.e. since Williams et al. (2014)), respectively, expanding the northern edges of its range. Expansion of the range of some bumble bee species has been linked to climate change in Europe (see Rasmont et al. (2021)), specifically warming winters (Biella and Galimberti 2020, Biella et al. 2020); Ghisbain et al. (2021) recently summarised the factors that could contribute to the spread of pollinators, including bumble bees, these also being linked to the climate (including heat tolerance), but also dietary flexibility. There is at least one older report on *B.bimaculatus* from Nova Scotia in the 1970s (Vander Kloet 1977), though the identity has not been confirmed, but it seems dubious. At least two of the pre-2000 records from Oregon (both from 1931) were identifed by Theodore Frison in 1932 (GBIF.org 2023), though as indicated above, these are likely misidentified (and see Koch et al. (2015)). As such, it will likely be important to monitor the spread of *B.bimaculatus* in Canada and the United States outside of its historic range and detemine if its arrival is impacting the established bumble bee fauna (e.g. Biella and Galimberti (2020), Ghisbain et al. (2021)). Equally important will be the continued digitisation and verification of historic collections from museum collections; it is quite possible that some species may be found outside their documented ranges (e.g. Klymko and Sabine (2015)).

Other bumble bee species have also expanded their ranges, including *B.vosnesenskii* within southern British Columbia (Fraser et al. 2012) which is likely also by natural means, though the occurrence of *B.impatiens* in British Columbia (Ratti and Colla 2010), Alberta (Palmier and Sheffield 2019), Manitoba (Gibbs et al. 2023) and Maritime Canada (Sheffield et al. 2003) and Newfoundland (Hicks et al. 2018) since the 1990s is likely due to the use of commercially available colonies in Canada (e.g. Whidden (1996), Van Westendorp and McCutcheon (2001)). Under both scenarios (i.e. natural spread and introduction), the impacts that non-native and recently-arriving native bumble bee species may have on local populations is of concern and should be monitored.

## Supplementary Material

XML Treatment for Bombus (Pyrobombus) bimaculatus

## Figures and Tables

**Figure 1a.**
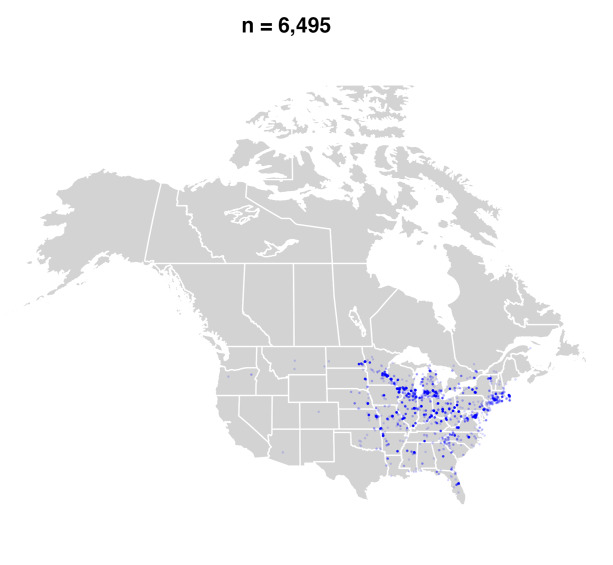
Pre-2000;

**Figure 1b.**
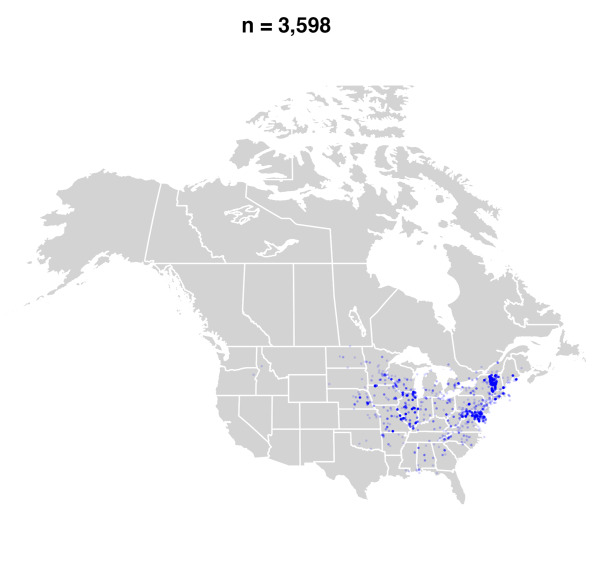
2000-2014;

**Figure 1c.**
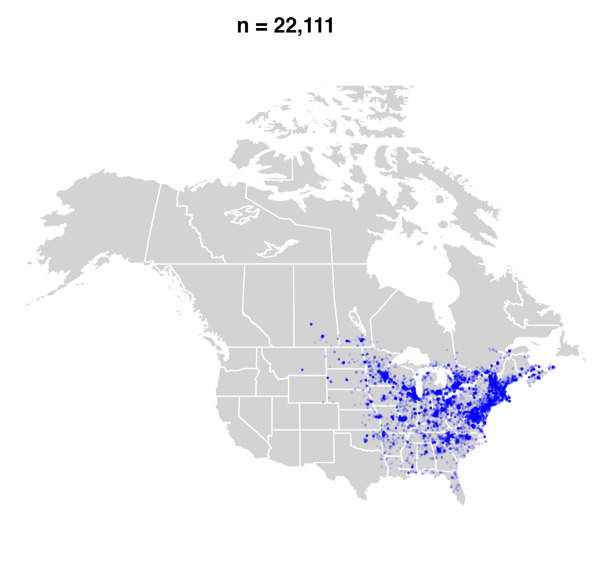
2015-present.
